# Immunometabolic Memory in Obesity: Nutritional Drivers, Persistence, and Reversibility

**DOI:** 10.3390/ijms27219666

**Published:** 2026-10-28

**Authors:** Claudia Reytor-González, Raynier Zambrano-Villacres, Nelly Andrade Mejia, Samuel Duran-Agüero, Daniel Simancas-Racines

**Affiliations:** 1 Center for Evidence Ecosystems, Implementation Science, and Decision-Making (CIDES), Facultad de Ciencias de la Salud y Bienestar Humano, Universidad Tecnológica Indoamérica, Ambato 180150, Ecuador; 2 Facultad de Ciencias de la Salud y Desarrollo Humano, Universidad ECOTEC, Samborondón EC092302, Ecuador; 3 Escuela de Posgrado, Doctorado en Ciencias de la Salud, Instituto Universitario Italiano de Rosario (IUNIR), Rosario S2000CTT, Argentina; 4 Facultad de Ciencias de la Salud, Universidad Espíritu Santo, Samborondón 0901952, Ecuador; 5 Escuela de Nutrición y Dietética, Facultad de Ciencias de la Rehabilitación y Calidad de Vida, Universidad San Sebastián, Providencia 7500000, Chile

**Keywords:** obesity, nutrition, immunometabolism, trained immunity, adipose tissue, weight loss, epigenetic memory, inflammation

## Abstract

Obesity reshapes immune function across adipose tissue, circulation, and hematopoietic compartments. Weight reduction improves cardiometabolic health, yet emerging evidence indicates that prior obesity can leave persistent cellular, transcriptional, and epigenetic changes. Whether nutrition can reverse this biological imprint remains uncertain. We conducted a critical narrative review informed by structured searches of PubMed/MEDLINE, the Cochrane Library, and Epistemonikos, complemented by backward and forward citation tracking. We prioritized mechanistic human studies, controlled dietary interventions, longitudinal weight-loss studies, and experiments that tested persistence after stimulus withdrawal or response to re-exposure. Evidence was interpreted according to biological compartment and study design rather than pooled statistically. Obesogenic exposures can alter innate immune metabolism, inflammasome signaling, hematopoiesis, and chromatin states. Animal studies provide the strongest evidence that these changes can persist after weight normalization and amplify responses during weight regain. Human studies show incomplete normalization of adipose immune states, persistent adipocyte programs, and durable methylation changes in T lymphocytes after weight loss. Dietary interventions can lower inflammatory tone and modify immune metabolism, microbial metabolites, and lipid mediator pathways, but no dietary pattern has been shown to erase established obesity-associated immune memory in humans. An evidence map built on a core set of 47 studies showed that experimental work dominates the induction, persistence and re-challenge domains, whereas human evidence clusters in post-weight-loss remodelling and dietary modulation; human re-challenge data were absent and no study provided a direct test of reversal in humans. Obesity-associated immunometabolic memory appears to be distributed across hematopoietic, immune, adipose, stromal, and microbial compartments. Nutrition can modulate mechanisms implicated in this persistence, but human evidence of reversal is lacking, and whether pharmacologically induced weight loss leaves a comparable imprint has not been tested. Mechanistic trials should separate energy deficit, diet composition, adiposity change, and weight-loss modality and include a weight-stable follow-up and functional re-challenge where feasible.

## 1. Introduction

Obesity alters metabolic regulation through changes that extend well beyond energy storage [1]. Early human and experimental studies established that expanding adipose tissue accumulates macrophages and develops a proinflammatory milieu associated with insulin resistance [2,3]. Adipose expression of tumour necrosis factor alpha provided one of the first mechanistic links between excess adiposity and impaired insulin action [4]. Subsequent work placed these observations within a broader model in which adipose tissue functions as an endocrine and immune organ whose cellular composition and secretory profile change as adiposity increases [5,6]. Chronic metabolic inflammation also intersects with pathways involved in type 2 diabetes, although the magnitude and biological relevance of this inflammatory response vary among individuals [7]. Visceral and subcutaneous adipose depots differ in cellular composition, vascular drainage and metabolic risk, which further limits simple inferences based on body mass alone [8].

Adipose inflammation is not a uniform macrophage response. Human studies have linked insulin resistance to macrophage-rich, fibrotic adipose tissue and to impaired tissue vascularization [9,10]. Adipose tissue also retains substantial structural and functional plasticity across changes in energy balance [11]. Single-cell analyses have revealed multiple macrophage, lymphoid, endothelial and stromal states that vary by depot, metabolic phenotype and degree of obesity [12,13,14]. These findings have weakened binary classifications of immune activation and shifted attention toward tissue niches, cell-state transitions and intercellular signaling. They also indicate that improvement in a circulating inflammatory marker cannot, by itself, establish normalization of the cellular programs that developed during obesity.

Weight loss improves many cardiometabolic and inflammatory outcomes, but the biological trajectory after weight reduction is less well defined [15]. Recent molecular studies suggest that some obesity-associated states persist after substantial weight loss. Adipose tissue from weight-reduced humans retains transcriptional features associated with prior obesity, while mouse adipocytes can preserve epigenetic marks after weight normalization [16]. Single-cell profiling has also shown selective, rather than complete, remodeling of the human adipose niche after therapeutic weight loss [17]. More recently, obesity-associated DNA-methylation patterns have been reported in human CD4-positive T lymphocytes after weight loss, extending the persistence signal beyond adipocytes and innate immune cells [18]. These observations support the existence of residual biological states after obesity, but they do not establish that such states are permanent, clinically harmful or responsible for subsequent weight regain.

This uncertainty is especially important in nutritional immunology. Obesity-associated immune dysfunction involves altered nutrient sensing, cellular metabolism, inflammatory signaling and communication between immune and metabolic tissues [19]. Trained immunity provides one mechanistic framework for persistent innate responses, but the term should be reserved for memory-like functional reprogramming demonstrated after an initial stimulus, a resting phase and a subsequent challenge [20]. Persistent inflammation, adaptive immune memory, adipocyte epigenetic memory, stromal remodeling and trained innate immunity therefore cannot be treated as interchangeable phenomena. Likewise, a reduction in C-reactive protein, cytokines or immune-cell abundance after dietary intervention indicates lower inflammatory activity, not necessarily reversal of an established cellular program. The central gap is therefore not whether nutrition can modify inflammation, but whether nutritional exposure can prevent, attenuate or reverse durable obesity-associated immunometabolic states, and under what experimental conditions such a claim would be justified.

A critical synthesis is needed because the relevant evidence is distributed across experimental immunology, adipose-tissue biology, nutritional intervention studies and longitudinal weight-loss research. The field also faces a recurrent inferential problem: findings from acute nutrient exposure are often used to explain long-term biological memory, while reductions in inflammatory biomarkers are sometimes interpreted as evidence of cellular resetting. This review addresses those distinctions by separating induction, persistence, re-challenge and reversal, and by comparing evidence across in vitro, animal and human studies. It also considers whether observed post-weight-loss states are better explained by durable cellular reprogramming, continued tissue signals, treatment context or incomplete recovery. Accordingly, this critical narrative review aims to synthesize and critically examine current evidence on persistent immunometabolic alterations associated with obesity, focusing on their nutritional drivers, persistence after weight loss, and potential reversibility.

## 2. Methodology

We conducted a critical narrative review informed by a structured literature search, following established methodological recommendations for narrative reviews [21,22]. PubMed/MEDLINE, the Cochrane Library, and Epistemonikos were searched from inception to 6 September 2026 using database-adapted combinations of controlled vocabulary and free-text terms related to obesity, adipose tissue, weight loss, weight cycling, immunometabolism, trained immunity, immune memory, epigenetic reprogramming, immune-cell populations, and nutritional exposures. Reference lists of relevant studies and reviews were additionally screened to identify complementary evidence. The authors assessed titles, abstracts, and full texts, selecting studies according to their relevance to the review objectives and resolving selection decisions through consensus. Evidence from in vitro and ex vivo experiments, animal models, human observational studies, and interventional studies was critically synthesized, considering mechanistic consistency, biological plausibility, study design, and translational relevance. To characterize the distribution of mechanistic evidence, we developed an evidence map comprising five biological domains: induction or training, persistence following stimulus withdrawal, re-challenge or weight cycling, diet-sensitive modulation, and post-weight-loss remodelling or reversal. Primary studies were classified according to their mechanistic contributions and study designs, allowing multiple domain assignments when supported by distinct findings. Complete search strategies, the synthesis framework, study-level classifications, bibliographic verification, and design-specific criteria for evidence interpretation are provided in Supplementary Figure S1 and Supplementary Tables S1–S5.

## 3. Nutrient Sensing and the Emergence of Immunometabolic Memory

Trained immunity provides a useful mechanistic framework, but it requires precision [23,24]. Innate immune memory describes altered secondary responses after a primary challenge, whereas trained immunity usually refers to durable functional reprogramming of innate cells supported by metabolic and epigenetic changes [23,24]. Foundational work showed that monocytes can acquire enhanced heterologous responses after microbial exposure and that these responses persist after the initiating stimulus has disappeared [25]. This concept differs from adaptive antigen-specific memory and from chronic stimulation, where the initiating signal remains continuously present [23,24]. Metabolism is not simply a consequence of immune activation; it helps determine cell state [26]. Trained monocytes increase glycolytic flux through mammalian target of rapamycin and hypoxia-inducible factor-1alpha signaling, while glutaminolysis and accumulation of tricarboxylic-acid-cycle intermediates can influence chromatin-modifying enzymes [26]. Histone methylation and acetylation then stabilize accessible regulatory regions that facilitate a different response to a later stimulus [27,28,29]. These experiments established a mechanistic chain from substrate use to chromatin state to immune function rather than a loose association between metabolism and cytokine production [27,29]. The operational criteria used to distinguish reduced inflammatory tone, cellular remodeling, persistent alteration, trained immunity, and reversal are summarized in Supplementary Table S5. These terms are not interchangeable and are used hierarchically throughout. Obesogenic memory is the umbrella term for any durable, obesity-associated alteration that outlasts the obese state, encompassing adipocyte epigenetic memory, adaptive lymphocyte memory, progenitor-level reprogramming and stromal or extracellular-matrix remodelling. Immunometabolic memory refers to the subset of those alterations that reside in immune cells and are accompanied by metabolic and chromatin changes. Trained immunity is the narrowest term and is applied only to innate cells or their progenitors in which reprogramming has been shown to persist through a rest phase and to alter the response to a subsequent stimulus.

Sterile metabolic stimuli can engage similar machinery [30,31]. Oxidized low-density lipoprotein produces prolonged proinflammatory reprogramming in human monocytes, and mevalonate-pathway activity can couple cholesterol synthesis to trained-immunity phenotypes [30,31]. Most directly relevant to obesity, a Western-style diet induced NLRP3-dependent innate immune reprogramming in mice, with persistent changes in myeloid progenitors despite withdrawal of the diet [32]. This study is central because it moves the concept from microbial training toward nutrition-driven sterile inflammation [32]. The persistence of innate programs requires an explanation beyond short-lived circulating monocytes [33,34,35]. Experimental and human vaccination studies show that hematopoietic stem and progenitor cells can undergo transcriptional and epigenetic remodeling and subsequently generate altered myeloid progeny [33,34]. Although these studies used microbial training rather than obesity, they establish the biological plausibility of a marrow-level reservoir [35,36]. Obesity itself can skew hematopoietic stem-cell output toward myelopoiesis, providing a parallel route by which sustained nutrient excess may influence future innate-cell composition [37].

Inflammasome signaling is one bridge between nutrient overload and altered innate function [38]. The NLRP3 inflammasome contributes to obesity-associated inflammation and insulin resistance in mice, and saturated fatty acids can promote inflammasome activation under metabolically stressed conditions [38,39]. Yet the common statement that Toll-like receptor 4 directly recognizes saturated fatty acids is too simplistic [40]. Careful mechanistic work indicates that Toll-like receptor 4 can mediate lipid-induced inflammation by reshaping macrophage metabolism without functioning as a conventional saturated-fatty-acid receptor [40]. Obese adipose tissue also generates a metabolic environment that differs from canonical microbial activation [41]. Macrophages exposed to combinations of glucose, insulin and palmitate develop a metabolically activated phenotype that cannot be described adequately by the classical M1/M2 axis [41]. Hyperglycaemia can leave a proinflammatory memory in innate cells, and recent work has tested how obesity-associated factors interact with beta-glucan training in human monocytes [42,43]. These findings reinforce a continuum between nutrient exposure and immune reprogramming but also expose a methodological problem: persistent activation produced by continued metabolic stress is not equivalent to memory demonstrated after a rest period [42,43].

Diet-derived molecules can also induce or oppose trained states [44]. Oat-derived beta-glucans have induced training-like metabolic reprogramming in experimental monocyte/macrophage systems, whereas omega-3 fatty acids signal through free-fatty-acid receptor 4 to suppress inflammatory macrophage pathways and improve insulin sensitivity in obese mice [44,45]. The ketone body beta-hydroxybutyrate inhibits NLRP3 activation, showing how a nutrient-derived metabolite can alter a pathway implicated in obesity-associated innate activation [46]. These observations establish mechanistic capacity; they do not prove that an omega-3-rich or ketogenic diet erases pre-existing obesity memory in humans [45,46]. Resolution biology provides another layer [47,48,49,50]. Resolvin D1 reduces inflammatory macrophage accumulation and improves insulin sensitivity in obese diabetic mice, and specialized pro-resolving lipid mediators can shift macrophage programs toward resolution [47]. Human visceral adipose tissue from obesity also responds ex vivo to resolvin signaling [48,49,50]. These findings are relevant because successful reversal may require active resolution rather than mere withdrawal of an inflammatory substrate; however, most studies assess acute pathway modulation rather than durable post-intervention memory [47,50].

Microbiota-derived metabolites broaden the nutritional signal beyond nutrients absorbed directly from food [51,52]. Short-chain fatty acids regulate regulatory T-cell homeostasis, and butyrate can promote regulatory T-cell differentiation partly through histone acetylation [51,52]. Related microbial metabolites can therefore connect diet, intestinal ecology and epigenetic regulation [53,54]. High-fat feeding can increase metabolic endotoxaemia in mice, while microbiota-directed experiments show that the intestinal ecosystem can transmit or sustain metabolic inflammatory phenotypes [55]. The same axis illustrates why mechanistic claims need careful calibration [56,57]. Emulsifiers can promote microbiota-dependent inflammation and metabolic syndrome in mice, and obesity-associated microbial configurations may persist after dieting and accelerate weight regain in experimental models [56,57]. Ketogenic diets alter the human and murine microbiome and can reduce intestinal T helper 17 cells, while a controlled human comparison of vegan and ketogenic diets produced sharply different peripheral immune signatures [58,59]. Fermented-food and high-fibre interventions also modulate microbiota and immune measures in humans [59,60]. None of these studies, however, demonstrate complete erasure of a pre-existing obesity-associated immune program.

Nutrient sensing can influence inflammatory signaling, intermediary metabolism, chromatin regulation and progenitor output [43,46]. The evidence is strongest for the ability of discrete stimuli to induce durable innate programs and for the ability of diet to alter immune-metabolic pathways [32,60]. The crucial inferential gap lies between these observations: a pathway that can be nutritionally modulated is not automatically a pathway whose established memory can be nutritionally reset [23]. Persistence after removal of obesity or obesogenic exposure should therefore be distinguished from acute anti-inflammatory efficacy [23].

**Figure 1:**
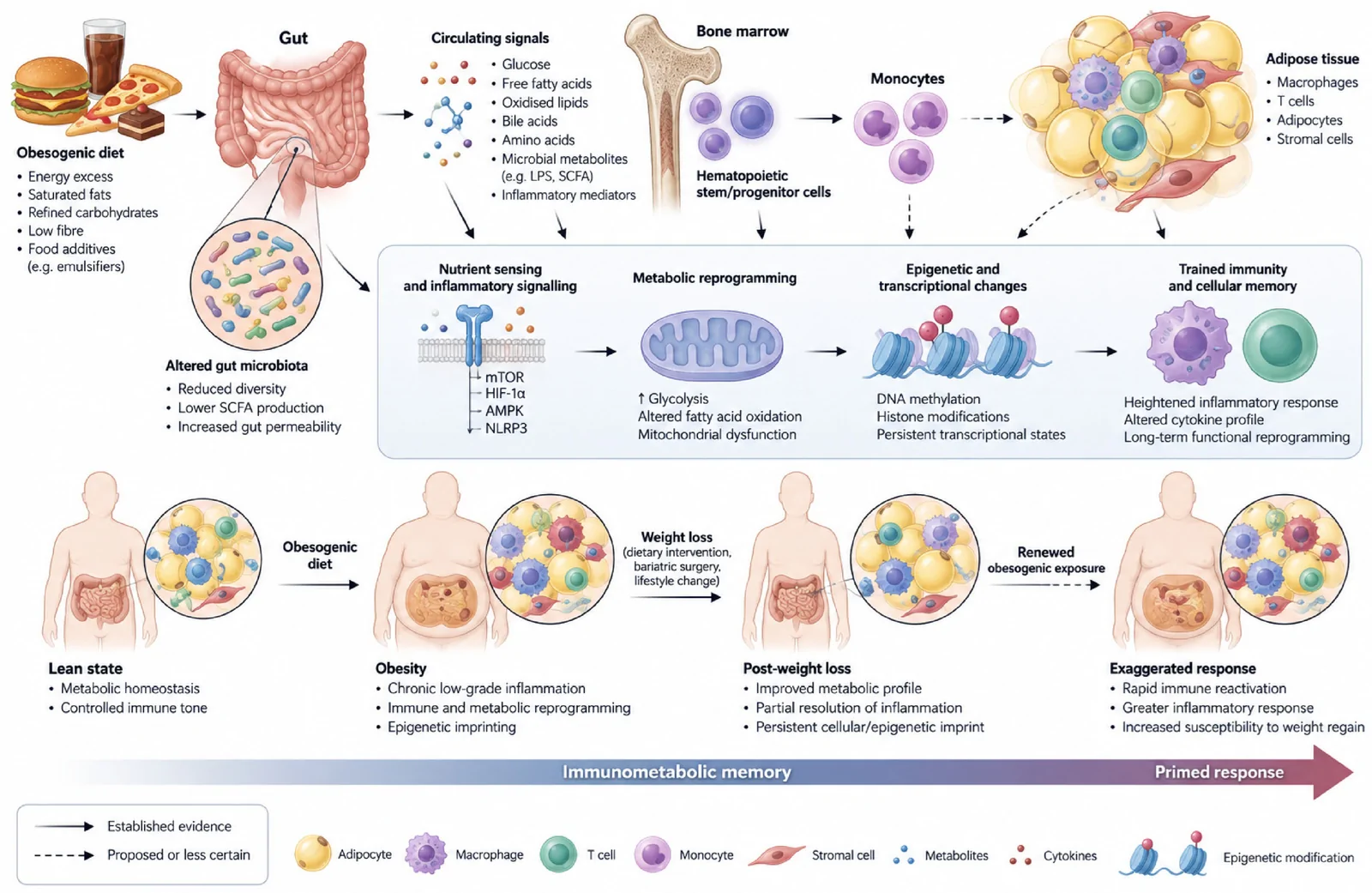
Nutritional exposures and the distributed architecture of immunometabolic memory. Obesogenic dietary patterns can alter gut-derived signals, circulating metabolites, hematopoietic progenitors and adipose-tissue immune states through nutrient sensing, metabolic reprogramming, inflammatory signaling and epigenetic regulation. Solid arrows denote relationships supported by experimental evidence; dashed arrows denote proposed or incompletely established links. The lower trajectory distinguishes obesity, weight loss and renewed obesogenic exposure to illustrate how a residual cellular imprint could amplify subsequent responses. The scheme is grounded in mechanistic evidence from trained-immunity, adipose-tissue and diet studies [25,26,27,28,29,30,31,32,33,34,35,36,37,38,39,40,41,42,43,44,45,46,47,48,49,50,51,52,53,54,55,56,57,58,59,60,61,62]. Abbreviations: AMPK, AMP-activated protein kinase; HIF-1α, hypoxia-inducible factor-1alpha; LPS, lipopolysaccharide; mTOR, mammalian target of rapamycin; NLRP3, NOD-, LRR- and pyrin domain-containing protein 3; SCFA, short-chain fatty acid; DNA, Deoxyribonucleic acid. Created in BioRender. Reytor, C. (2026) https://BioRender.com/c2pqbuo.

A second inferential issue concerns dose and duration [59]. Acute exposure to glucose, fatty acids, ketones or microbial metabolites can alter signaling within minutes or hours, whereas durable reprogramming requires enough time to change transcriptional networks, organelle function or chromatin architecture [30,32,39]. Obesity develops over much longer periods than most controlled-feeding experiments, and repeated postprandial exposures may produce a different biological state from continuous high-dose stimulation in cell culture [30]. Translational claims should therefore privilege studies that model exposure history explicitly and should treat short-term nutrient challenges as evidence of pathway responsiveness rather than memory itself [26]. Figure 1 integrates these nutritional signals with the cellular compartments in which persistent reprogramming may arise.

## 4. Where Does Obesity-Associated Immune Memory Reside?

Adipose tissue is an obvious candidate reservoir because it is both a nutrient-storage organ and an immune niche [61,62,63,64]. Human adipocytes can promote monocyte recruitment, and dead hypertrophic adipocytes are surrounded by macrophage-rich crown-like structures [61,62]. In diet-induced obesity, macrophage number and phenotype change markedly, with chemokine pathways such as monocyte chemoattractant protein-1 contributing to recruitment [63,64]. These findings place macrophages at the interface between tissue injury, lipid handling and inflammation, but macrophage accumulation alone does not establish memory. Macrophage heterogeneity complicates interpretation further [65,66,67]. CD11c-positive macrophage populations have distinct metabolic and inflammatory functions in obese mice, while human CD11c-positive/CD206-positive adipose macrophages are associated with insulin resistance and can impair insulin-stimulated glucose uptake in adipocytes [65,66]. Human obesity also contains individuals with relatively low adipose inflammation despite comparable adiposity, supporting the view that tissue immune state is not reducible to body mass alone [67].

Adaptive lymphocytes form a second candidate reservoir [68,69,70]. CD8-positive effector T cells can precede macrophage recruitment during diet-induced obesity, whereas lean visceral fat contains a distinctive regulatory T-cell population that is depleted with obesity [68]. Peroxisome proliferator-activated receptor gamma is central to the phenotype and accumulation of adipose regulatory T cells, linking lipid-sensing transcriptional programs to adaptive immune homeostasis [69,70]. These studies establish that adipose T-cell composition responds to metabolic state and can influence insulin sensitivity. B cells add another form of adaptive regulation [71,72,73]. In obese mice, B cells can promote insulin resistance through T-cell modulation and pathogenic immunoglobulin G responses, while a distinct interleukin-10-producing regulatory B-cell compartment restrains adipose inflammation [71]. B-1a cells can also attenuate insulin resistance [72,73]. This functional diversity makes it implausible to speak of a single ‘adaptive immune response’ to obesity; cell lineage, differentiation state and tissue localization matter.

Type 2 immune cells contribute to homeostatic adipose biology [74,75,76,77]. Eosinophils support alternatively activated macrophages and glucose homeostasis, while eosinophil and type 2 cytokine signaling can promote beige-fat development in mice [74,75]. Group 2 innate lymphoid cells also regulate beiging and limit obesity in experimental models [76,77]. These pathways show that immune cells participate in normal adipose adaptation, not only in pathology, and therefore an apparent post-weight-loss change may represent physiological remodeling rather than residual inflammatory memory. Other immune populations broaden this network [78,79,80,81]. Mast-cell deficiency or stabilization reduces diet-induced obesity and glucose intolerance in mice, and neutrophil elastase contributes to high-fat-diet-associated insulin resistance [78]. Dendritic cells accumulate in obese adipose tissue, can promote T helper 17 responses, and have functions that are separable from those of macrophages [79,80]. These studies establish early and diverse immune participation, but persistence after weight loss has been studied far less consistently across these cell types [81].

Natural killer T cells illustrate the danger of assuming uniform directionality [82,83,84,85]. Adipose invariant natural killer T cells can protect against metabolic dysfunction through regulatory cytokines, yet chronic activation by lipid excess can promote inflammation in other experimental settings, and type II natural killer T cells can aggravate diet-induced disease [82,83]. Human visceral adipose natural killer cells also associate with low-grade inflammation and insulin resistance [84,85]. The immune consequences of obesity therefore depend on subset, activation state, duration and tissue context. Single-cell approaches have exposed macrophage states that are particularly relevant to memory [86,87,88]. CD9-positive macrophages localize to crown-like structures and differ functionally from other adipose macrophages, while TREM2-positive lipid-associated macrophages emerge in obese adipose tissue and participate in lipid handling [86,87]. Stromal cells also coordinate immune-cell recruitment and adipocyte generation [88]. Persistent obesity-associated states may therefore be stored as properties of an ecosystem rather than a single lineage.

Tissue hypoxia and antigen presentation provide additional mechanisms that could maintain local states [89,90]. Experimental obesity increases adipose hypoxia and cytotoxic T-cell infiltration, and an MHC class II-dependent feedback loop between macrophages and CD4-positive T cells can sustain adipose inflammation [89]. Such reciprocal circuits can generate persistence without requiring permanent epigenetic marks in every cell [90]. Distinguishing cell-intrinsic memory from niche-maintained reinforcement is therefore essential. The most defensible model is distributed [17]. Bone marrow may preserve altered progenitor output; circulating monocytes and lymphocytes can carry durable transcriptional or epigenetic programs; adipose immune cells can maintain local activation; adipocytes can retain their own epigenetic history; and stromal, vascular and extracellular-matrix changes can recreate signals that reinforce those cell states [16,18,35,37]. This distributed model better accommodates current human data than a macrophage-only model and predicts that successful reversal may require normalization at several biological levels [87].

Adipocytes themselves deserve particular attention because they outlive many infiltrating immune cells and directly determine the lipid, endocrine and mechanical environment of the niche [11,17,61,64]. The epigenetic persistence described after obesity means that a post-weight-loss adipocyte may not be equivalent to an adipocyte that has never experienced hypertrophic stress [16]. If such adipocytes respond differently to renewed nutrient excess, they could re-create chemotactic or inflammatory conditions that recruit newly generated immune cells even if those immune cells carry no intrinsic memory [16]. This possibility provides a parsimonious explanation for some tissue-level persistence and reinforces the need to distinguish immune-cell memory from memory of the broader adipose organ [16].

The marrow–blood–adipose axis also creates a hierarchy of possible persistence [33,35,86,87]. A progenitor-level program can influence successive generations of circulating myeloid cells [18,37]. A circulating lymphocyte can carry epigenetic information between tissues [18,37]. And a remodeled adipose niche can select or reinforce particular incoming cell states [18,37]. These mechanisms are not mutually exclusive [87]. Their relative importance probably differs across the induction, active weight-loss and weight-stable phases, which helps explain why studies sampling different compartments or time points can appear inconsistent while still describing different parts of the same biological process [87]. This hierarchy also predicts that reversal may occur unevenly [17,67]. A dietary intervention could normalize circulating metabolites rapidly while leaving marrow output unchanged [16,18]. Adipose macrophage abundance could fall while residual T-cell or stromal programs persist [16,18]. Or an adipocyte epigenetic program could remain despite substantial immune-cell turnover [16,18]. A convincing study of recovery therefore needs to define which compartment is expected to normalize and on what timescale [35]. Without that specification, apparently conflicting results may simply reflect different biological clocks rather than true disagreement [67].

## 5. Weight Loss Is Not Necessarily an Immunological Reset

Human weight-loss studies established long ago that adipose inflammation is modifiable [91,92,93,94]. Very-low-energy diets alter large sets of inflammation-related adipose transcripts, surgery-induced weight loss reduces macrophage infiltration and chemotactic gene expression, and combined diet and exercise lowers systemic inflammatory markers and adipose macrophage markers [91]. Dietary weight loss can also reduce macrophage content in subcutaneous adipose tissue [92,93,94]. These findings are clinically important, but they show that inflammation can decrease. They do not establish that previous obesity leaves no residual biological trace. Weight loss itself can provoke a transient immune response because mobilization of stored lipid changes the adipose microenvironment [95]. In mice, lipolysis during weight reduction causes dynamic immune-cell recruitment, which means that sampling time can reverse the apparent direction of an effect [95]. In humans with obesity and type 2 diabetes, very-low-calorie diets change inflammatory gene expression in both adipose tissue and peripheral monocytes [96]. Consequently, an early post-intervention biopsy may capture remodeling rather than a stable post-obesity state.

Energy deficit and macronutrient composition are also difficult to disentangle [97,98]. In controlled hypocaloric interventions, adipose gene-expression changes often track energy restriction and fat-mass loss more strongly than the dietary fat-to-carbohydrate ratio [97]. Transcriptomic differences during weight maintenance have been associated with subsequent weight regain, including programs related to cellular proliferation and mitochondrial oxidative phosphorylation [98]. These studies support persistent tissue heterogeneity, but they cannot by themselves determine whether the relevant signals are immune memory, altered adipocyte biology or a consequence of ongoing energy balance. Bariatric surgery adds another layer of complexity because it changes energy intake, gut anatomy, bile-acid signaling and hormones simultaneously [99,100]. Longitudinal human work shows that glucose control can improve rapidly while adipose leukocyte infiltration and inflammatory-gene expression remain abnormal for longer [99]. In a randomized calorie-restriction study in women with overweight or obesity, improved insulin sensitivity did not equate to complete normalization of adipose immune-cell composition or cytokine patterns [100]. These discordances are more informative for the memory question than studies measuring only weight and C-reactive protein.

Experimental reversal studies provide stronger evidence of persistence [101,102,103]. Macrophage proliferation can sustain adipose inflammation in formerly obese mice, and macrophage-dependent CD8-positive T-cell inflammation can remain active during weight loss despite normalization of several metabolic variables [101]. When caloric restriction is imposed while a high-fat dietary composition is maintained, weight loss attenuates but does not fully normalize visceral adipose inflammation [102,103]. These models demonstrate that normalization of body mass is not sufficient to guarantee normalization of immune state. Several independent mouse models now support the broader concept of obesogenic memory [104,105,106]. Persistent changes in regulatory T cells, innate lymphoid cells, macrophages and adipocytes have been reported after body weight normalization [104]. CD4-positive T cells from previously obese mice can transfer an enhanced weight-regain phenotype to immune-deficient recipients, providing functional evidence that an adaptive immune compartment can carry obesity history [105,106]. Multi-omic profiling further shows that obesity-associated adipose immune phenotypes can persist through weight loss and worsen again with weight regain.

Macrophage training and adaptive memory may cooperate rather than compete as explanations [107,108]. In mouse models, weight cycling has been associated with trained-immunity features in adipose macrophages, while a 2026 intervention study found that disrupting the CD70-CD27 axis reduced memory T-cell accumulation and attenuated the exaggerated glucose intolerance observed after weight cycling [107]. These experiments provide comparatively strong mechanistic evidence because they manipulate memory-related pathways rather than only documenting persistence [108]. Their limitation is equally important: they are mouse studies, and weight cycling protocols impose dietary transitions that do not reproduce all forms of human weight loss. CD8-positive effector-memory T cells have been proposed as a principal cellular carrier of obesogenic memory in white adipose tissue, with the CD70-CD27 costimulatory axis identified as the pathway through which that memory is established [109]. This is consistent with the observation that CD70 deletion leaves the metabolic response to stable obesity largely intact while protecting against the worsened glucose tolerance that follows weight cycling [108], which localizes the deficit to memory formation rather than to obesity itself. A dedicated review of immune-cell-mediated memory in weight cycling reaches a convergent conclusion across macrophages, CD4-positive and CD8-positive T cells, regulatory T cells and monocyte subsets [110]. The role of CD8-positive T cells in initiating adipose inflammation during obesity [68] is a separate claim from their memory phenotype after weight loss, and it is the latter that the weight-cycling literature now supports.

Other tissue features may predict recurrence without being immune memory in the strict sense [111]. Leukocyte integrins and extracellular-matrix remodeling have been associated with risk of weight regain after human weight loss, and partial weight regain in mice alters adipose immune and inflammatory markers [111,112]. These signals could act as a permissive niche that accelerates re-inflammation when energy surplus returns [111]. The distinction matters because a tissue can behave as if it ‘remembers’ obesity even when the persistence is maintained by extracellular architecture rather than stable cell-intrinsic chromatin marks [111].

The strongest human evidence now comes from molecular studies with repeated sampling [18]. Hinte and colleagues found persistent obesity-associated transcriptional signatures in human adipose tissue after substantial weight loss and epigenetic memory in mouse adipocytes [16,17]. Miranda and colleagues showed selective rather than complete remodeling of the human adipose niche after therapeutic weight loss [16,17]. Niven and colleagues extended persistence to DNA methylation in human CD4-positive T lymphocytes [18]. Together these studies make a complete-reset model difficult to sustain, but they do not show that the residual states inevitably cause weight regain or cardiometabolic events [18]. Human evidence now extends beyond adipose tissue and lymphocytes to circulating innate cells. In adults undergoing metabolic bariatric surgery, leukocyte counts and high-sensitivity C-reactive protein fell to the levels of normal-weight controls six months after surgery, whereas the hyperresponsive proinflammatory monocyte phenotype was only partially reverted, a dissociation mirrored in the monocyte transcriptional profile; exposure of healthy-donor monocytes to adipose tissue from patients with obesity induced a persistently augmented cytokine production capacity one week later [113]. This provides direct human evidence that an innate trained-immunity phenotype can outlast substantial weight loss and implicates adipose tissue itself as the inducing signal. Table 1 summarizes the studies that most directly support or challenge persistent immunometabolic memory, together with the species and evidence level of each.

We therefore propose immunometabolic hysteresis as a descriptive framework: the immune-metabolic state at a given body weight can depend on the path by which that weight was reached [15,17]. Hysteresis does not imply irreversibility [108]. It predicts only that recovery may follow a different trajectory from disease development and may lag behind changes in adiposity [108]. Demonstrating true hysteresis requires matched states, repeated measurements and ideally re-exposure experiments; otherwise, persistent differences may reflect age, treatment modality, diet composition, medication or incomplete weight stabilization rather than biological memory itself [16,106,108]. The human evidence still has important weaknesses [100]. Weight-loss cohorts are usually small, tissue availability is selective, and baseline obesity duration is rarely known with precision [17]. Bariatric cohorts also differ systematically from participants in diet-only trials, while medications for diabetes and cardiovascular disease can alter inflammatory pathways independently of weight loss [99,100]. For these reasons, persistence should be interpreted as a reproducible biological signal rather than proof that obesity creates an irreversible lesion [18]. The strongest inference emerges when different methods, including histology, flow cytometry, transcriptomics, epigenomics and functional assays, point in the same direction [100].

The concept of immunometabolic hysteresis shares important features with metabolic memory, particularly the persistence of biological consequences after the initiating metabolic exposure has changed. In diabetes, metabolic memory was established through the long-term follow-up of the Diabetes Control and Complications Trial (DCCT/EDIC), which demonstrated sustained benefits of early intensive glycaemic control despite subsequent convergence in glycaemic levels [114]. Experimental studies have further identified persistent inflammatory responses and epigenetic alterations in innate immune cells following hyperglycaemic exposure [42]. Within the context of obesity, immunometabolic hysteresis describes the possibility that the immune-metabolic state following weight loss remains dependent on prior exposure to an obesogenic environment, rather than being determined exclusively by current adiposity. This framework accommodates multiple, potentially interacting mechanisms, including cellular reprogramming, altered immune-cell composition, and persistent tissue remodelling, without attributing the observed phenotype to a single causal pathway. Its evaluation requires longitudinal and compartment-specific investigations comparing individuals with different obesity histories at comparable levels of adiposity, ideally following weight stabilization and incorporating functional re-challenge experiments to distinguish persistent alterations from altered responses to subsequent metabolic stimuli.

**Figure 2:**
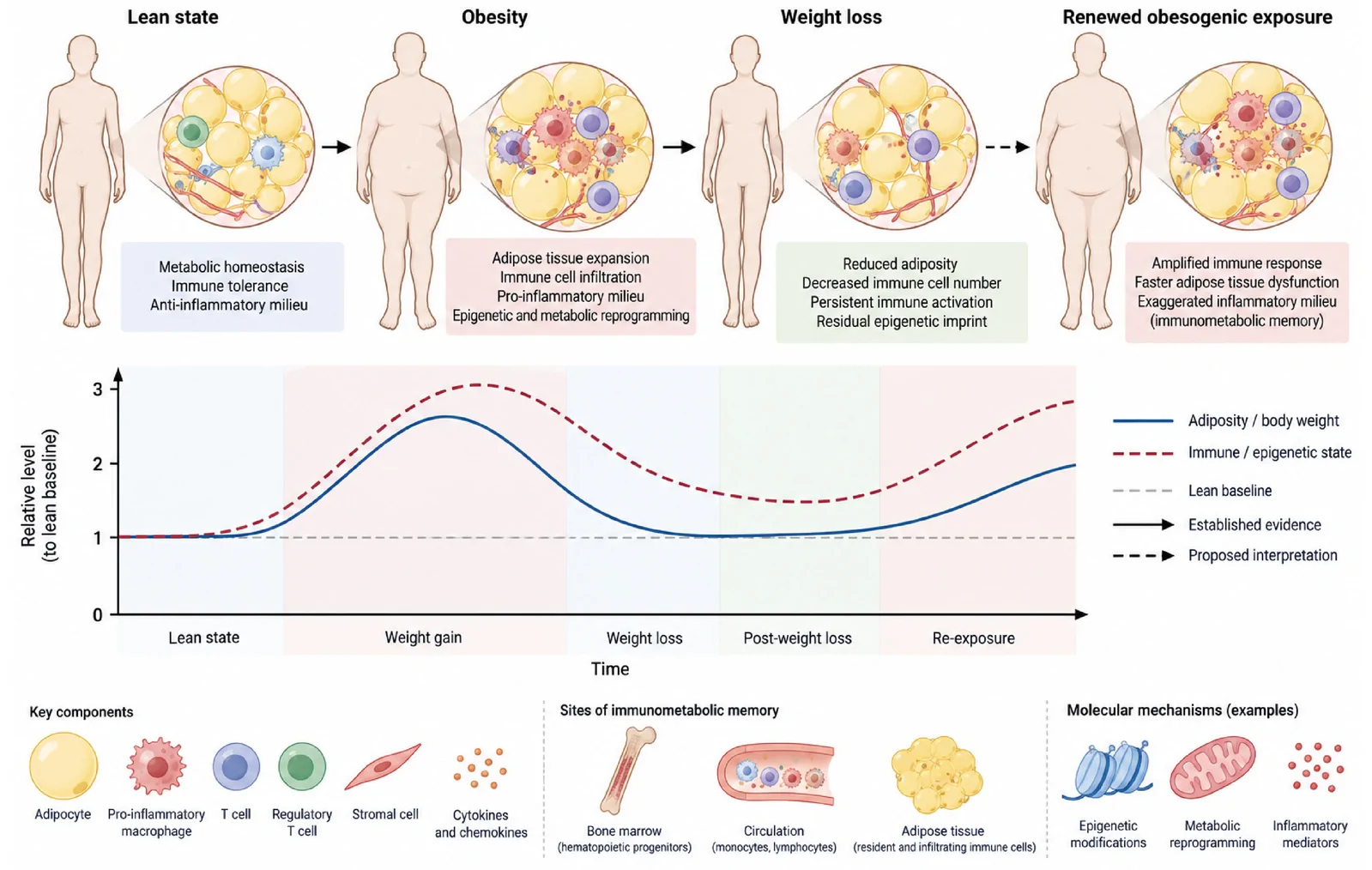
Immunometabolic hysteresis across obesity, weight loss and renewed exposure. Body weight and adiposity can improve faster than immune, stromal or epigenetic programs normalize. The conceptual separation between the adiposity trajectory and the immune/epigenetic trajectory illustrates a testable form of hysteresis: two individuals at a similar current body weight may occupy different biological states because one has a history of obesity. The concept is supported by post-weight-loss adipose, T-cell and weight-cycling studies but should not be interpreted as proof of permanent or universal memory [16,17,18,93,94,95,96,97,98,99,100,101,102,103,104,105,106,107,108,111,112,115,116]. Created in BioRender. Reytor, C. (2026) https://BioRender.com/2hj834r.

Recovery may also be compartment-specific [102]. Circulating inflammatory markers can normalize while adipose cellular states remain altered, and insulin sensitivity can improve before local immune remodeling is complete [95,99,100,102]. Conversely, transient leukocyte recruitment during lipolysis can make adipose tissue appear more inflamed even while systemic metabolic health improves [99]. These temporal and spatial dissociations explain why studies that use only blood biomarkers may overestimate biological resetting and why tissue sampling during active weight loss can underestimate it [102]. Figure 2 conceptualizes the resulting divergence between recovery of adiposity and recovery of immune or epigenetic state.

## 6. Can Nutrition Reprogram the Reprogrammed Immune System?

The capacity of nutritional interventions to modify established immunometabolic memory depends on the molecular mechanisms sustaining the altered cellular state. Epigenetic modifications differ in their dynamics and persistence, although their stability is determined by genomic context, cellular identity, and environmental conditions rather than by a fixed hierarchy of reversibility [117,118]. Histone acetylation is particularly responsive to metabolic signals through the availability of acetyl-coenzyme A and the opposing activities of histone acetyltransferases and deacetylases. Histone methylation can support more persistent regulatory states, including the maintenance of stimulus-responsive enhancers, whereas DNA methylation may contribute to longer-lasting transcriptional alterations. Nevertheless, these modifications remain potentially dynamic, and their responsiveness to nutritional exposures does not necessarily imply reversal of a previously established immunometabolic program. Cellular turnover introduces an additional dimension, as changes in immune-cell composition may modify tissue inflammatory profiles without reversing the intrinsic programming of individual cells [16,18,37].

Experimental studies provide evidence that established immune programs can retain persistent regulatory features while remaining susceptible to subsequent modification. Latent enhancers activated during macrophage stimulation may preserve regulatory marks after stimulus withdrawal, facilitating enhanced transcriptional responses upon re-exposure [119]. Conversely, β-glucan has been shown to partially reverse LPS-induced tolerance in human monocytes through epigenetic and transcriptional remodelling, with functional responsiveness also restored in monocytes obtained following experimental endotoxaemia [120]. These findings demonstrate that previously acquired immune states are not necessarily irreversible, although their relevance to obesity-associated immunometabolic memory remains to be established. Importantly, modifications in inflammatory activity, transcriptional responsiveness, and epigenetic architecture represent distinct outcomes that should not be interpreted interchangeably.

Nutritional exposures may influence these processes through several complementary pathways. Short-chain fatty acids, particularly butyrate, can regulate histone acetylation through histone deacetylase inhibition, influencing intestinal macrophage responses and regulatory T-cell differentiation [52,121]. However, these experimental findings do not establish whether dietary modulation of short-chain fatty acid availability can reverse persistent immune programming in adipose tissue. Omega-3 fatty acids and specialized pro-resolving mediators promote inflammatory resolution and tissue homeostasis [45,47,48], but resolution does not necessarily imply elimination of the molecular features underlying immune memory. Similarly, energy restriction and time-restricted eating influence metabolic substrate availability and cellular signalling pathways that interact with epigenetic regulation [26,29], whereas Mediterranean dietary patterns integrate multiple nutritional mechanisms that complicate the attribution of effects to specific molecular pathways [122,123,124]. Consequently, improvements in inflammatory biomarkers following dietary interventions cannot, by themselves, demonstrate reversal of established immunometabolic memory. Addressing this distinction will require longitudinal, cell-type-resolved studies combining functional assessments with locus-specific epigenetic profiling, ideally incorporating weight stabilization, appropriate comparison groups, and metabolic re-challenge. Such approaches could help determine whether nutritional interventions induce transient immunomodulation, durable functional remodelling, or genuine reversal of obesity-associated cellular programming.

Caloric restriction provides the clearest human evidence that nutrition can alter immune function independently of a disease-specific drug [115,116]. In adults with overweight, calorie restriction enhanced measures of T-cell-mediated immunity, and longer-term moderate restriction reduced systemic inflammatory markers without impairing cell-mediated immune responses in non-obese adults [115]. These trials show that nutritional energy flux can reshape immune physiology, but neither was designed to test whether obesity-associated cellular memory had been erased [116]. Epigenetic analyses from the CALERIE trial reinforce that distinction [125,126]. Two years of calorie restriction produced measurable changes in blood DNA-methylation patterns and modest effects on methylation-based measures of biological aging, yet these findings concern systemic aging-related signatures rather than reversal of obesity-specific immune memory [125]. A nutritional intervention can therefore alter the epigenome without demonstrating that a previously established obesogenic program has returned to a never-obese state [126].

Diet quality can modulate inflammation beyond simple calorie reduction [122,123,124]. In the PREDIMED trial, Mediterranean dietary patterns produced long-term changes in inflammatory and immune-related biomarkers, and mechanistic substudies found reduced activation of circulating immune cells [122,123]. Contemporary meta-analytic evidence generally supports modest reductions in selected inflammatory biomarkers with Mediterranean-style diets, although heterogeneity in populations, background diets and biomarker panels remains substantial [124]. These effects justify studying Mediterranean patterns as candidate modifiers of immunometabolic recovery, not describing them as proven memory-erasing interventions. Fermentable fibre offers a mechanistically attractive route because microbial short-chain fatty acids can influence regulatory T cells and chromatin acetylation [51,52,127,128]. The Food4Gut randomized trial reported changes in fecal and inflammatory markers after a prebiotic fibre intervention in obesity, and targeted colonic delivery of propionate has been tested for effects on body-weight regulation and adiposity [127,128]. Yet the translation from intestinal regulatory pathways to persistent adipose or hematopoietic memory remains largely inferential [127]. Future trials would need paired immune-cell epigenomics or functional re-challenge to establish that link [128].

Omega-3 fatty acids illustrate a similar gap between mechanism and human tissue evidence [45,47,48,129,130]. Experimental studies identify free-fatty-acid receptor 4 signaling and specialized pro-resolving lipid mediators as plausible routes to suppress macrophage inflammation and promote resolution [45,47,48]. In human obesity, randomized supplementation has altered inflammatory markers in visceral adipose depots, but another double-blind trial found limited modification of subcutaneous adipose inflammation [129,130]. This inconsistency is informative: changing a nutrient-sensitive pathway does not guarantee uniform remodeling across adipose depots or immune endpoints [129,130]. Food processing may influence the immune-metabolic environment through energy intake and the gut ecosystem [56,131]. A tightly controlled inpatient trial showed that an ultra-processed diet increased ad libitum energy intake and body weight compared with an unprocessed diet, while experimental emulsifier studies demonstrate microbiota-dependent inflammatory effects [131]. These studies support the importance of dietary context but do not isolate an immune-memory mechanism [56]. The same caution applies to observational associations between ultra-processed food exposure and inflammatory biomarkers, which are vulnerable to confounding by adiposity and overall dietary pattern.

Time-restricted eating and fasting alter nutrient timing, substrate use and circadian signaling [132,133]. Early time-restricted feeding improved insulin sensitivity and oxidative-stress measures in men with prediabetes even without weight loss, whereas shorter feeding windows can reduce body weight in adults with obesity [132]. Direct evidence that these approaches modify persistent adipose immune states is sparse [133]. Their value for the present hypothesis lies in the ability to change metabolic flux without necessarily changing diet composition, creating a useful experimental contrast for separating energy timing from nutrient quality and adiposity. Weight-loss modality further complicates nutritional interpretation [134,135]. Bariatric surgery can reprogram adipose inflammatory signaling and produces metabolic changes that precede complete inflammatory resolution [134]. Gut microbiota, bile acids and incretin responses change alongside food intake, making surgery biologically informative but causally complex [135]. Any comparison between surgery and dietary restriction therefore needs to distinguish common consequences of fat-mass loss from modality-specific effects on immune and microbial pathways.

Incretin-based pharmacotherapy has become a dominant modality of weight reduction and raises the memory question in a form that has not been studied. Whether weight loss driven by glucagon-like peptide-1 receptor agonism, or by dual glucose-dependent insulinotropic polypeptide and glucagon-like peptide-1 receptor agonism, leaves the same adipose immune imprint as dietary weight loss is unknown. Two considerations make the question tractable. First, discontinuation produces rapid and substantial weight regain, creating a re-exposure phase of the kind the memory framework predicts to be informative: in a randomized withdrawal trial, participants switched from tirzepatide to placebo after thirty-six weeks regained a large proportion of the weight they had lost, whereas those who continued treatment maintained and extended their reduction [136]; one year after withdrawal of semaglutide and its accompanying lifestyle intervention, participants had regained approximately two-thirds of their prior weight loss, with most cardiometabolic improvements reverting towards baseline [137]. These trajectories constitute pharmacologically induced weight cycling, and the experimental literature predicts that renewed obesogenic exposure after weight loss amplifies rather than merely restores adipose inflammation [106,107], a connection drawn explicitly in the study that disrupted the CD70-CD27 axis [108].

Second, these agents may act on the immune compartment directly as well as through fat-mass loss, so pharmacological and dietary interventions producing identical weight change need not produce identical immune outcomes. Glucagon-like peptide-1 receptor agonists have been reported to attenuate adipose tissue inflammation and to influence adipose browning, mitochondrial biogenesis and autophagy, but this evidence is overwhelmingly preclinical and has not been confirmed in humans [138]. Neither equivalence nor difference can therefore be asserted. Weight-loss modality should be treated as a distinct exposure within the analytic framework developed in Section 7, and the most informative near-term study would sample adipose tissue and circulating immune cells before treatment, after weight stabilization on treatment, and after a period of planned discontinuation, comparing the result with dietary weight loss of comparable magnitude.

Persistent microbial effects may also contribute to post-obesity biology [139,140,141,142]. Long-term exposure to obesity-associated microbiota can promote host insulin resistance in experimental systems, and lower gut microbial richness is associated with adverse metabolic markers in humans [139]. Caloric restriction can modify gut permeability, inflammation markers and fecal microbiota in women with obesity [140,141,142]. These observations make the microbiome a plausible component of distributed immunometabolic memory, but evidence that microbial normalization is necessary or sufficient for immune resetting remains incomplete [139,141]. The most useful conceptual distinction is between suppression, remodeling and erasure [20,125]. Suppression means that inflammatory outputs fall while the underlying cellular program may persist [100,129]. Remodeling means that cell composition, metabolism or chromatin changes substantially toward a healthier state [16]. Erasure is the strongest claim and would require demonstration that a post-obesity immune system behaves and responds comparably to a never-obese reference, including after a standardized challenge [18]. Existing nutritional trials provide substantial evidence for suppression and some evidence for remodeling; robust human evidence for erasure is not yet available [129].

This hierarchy changes how nutritional outcomes should be interpreted [130]. A lower C-reactive protein concentration after a Mediterranean diet supports anti-inflammatory activity [122,123,129,130]. A shift in macrophage abundance or transcription supports tissue remodeling [122,123,129,130]. A durable normalization of chromatin accessibility after weight stabilization would support deeper reprogramming [122,123,129,130]. And a normal response to a later standardized challenge would provide the strongest evidence that memory had been functionally extinguished [122,123,129,130]. Most published nutrition studies stop at the first or second level [123]. The absence of deeper endpoints is not a failure of those trials, which were designed for different questions, but it means they cannot be repurposed retrospectively as proof of memory reversal [130].

Nutritional strategies may ultimately work best by preventing reinforcement rather than erasing every molecular trace [47,57,107]. Reducing saturated-fat overload, improving dietary fibre supply, supporting production of pro-resolving lipid mediators and avoiding repeated large weight fluctuations could lower the probability that residual programs are repeatedly reactivated [40,51]. This preventive interpretation is more consistent with current evidence than the stronger claim that a particular food, supplement or dietary pattern can wipe an established epigenetic history clean [107]. The practical implication is that nutritional trials should avoid equating biological plausibility with demonstrated reversal [46,47]. Nutrient-sensitive receptors, inflammasomes, microbial metabolites and lipid mediators identify plausible intervention nodes, but a mechanistic target can be modifiable without being the limiting determinant of recovery [40,45,51]. The strongest candidates for future trials are therefore interventions with both a well-defined molecular pathway and evidence that the pathway remains abnormal after weight stabilization [51].

Exercise is not a nutritional exposure, but it dissociates immune remodelling from fat-mass loss more cleanly than any dietary intervention and is therefore informative here. Two weeks of either high-intensity interval or moderate-intensity continuous training in inactive adults with obesity altered leukocyte chemokine-receptor expression in the absence of any change in percentage body fat, visceral adipose tissue or circulating chemokines, and the two modalities moved different receptors in different directions [143]. Over a longer interval, a sixteen-week combined aerobic and resistance programme in postmenopausal women with obesity reduced proinflammatory and increased anti-inflammatory adipose tissue macrophage populations in paired subcutaneous biopsies [144]. The adipose immune compartment can therefore be shifted by a stimulus that is neither an energy deficit nor a change in diet composition, which means that physical activity must be measured, and ideally controlled, when post-weight-loss immune states are interpreted; most of the studies reviewed here did not do so.

Findings that do not fit the persistence model also require consideration. In rats, the loss of the anticontractile function of perivascular adipose tissue caused by diet-induced obesity was reversed by sustained weight loss, with restoration of adipocyte size, reduced local tumour necrosis factor alpha, recovered nitric oxide synthase function and normalization of circulating adipokines [145]. In humans, carotid arterial inflammation assessed by fluorodeoxyglucose uptake declined after bariatric surgery to values comparable with both lean and overweight control groups [146], and proteomic profiling of abdominal subcutaneous adipose tissue after surgical weight loss showed coordinated downregulation of immune-system pathways together with recovery of mitochondrial density [147]. These studies are not interchangeable with the persistence literature, since two are not immune-cell resolved, one is an animal model and none include a re-challenge, but they establish that some obesity-associated abnormalities normalize in some compartments after sustained weight loss. Read alongside the evidence for incomplete remodelling [17], they support a graded rather than a categorical conclusion: recovery is uneven across compartments and endpoints, and the compartment a study samples partly determines whether it appears to support persistence. A reporting asymmetry should also be acknowledged. A durable post-obesity difference is a readily publishable result, whereas complete normalization of an adipose immune population may be read as a negative finding and reported less prominently. Because the persistence literature remains small, and because several of its most-cited findings originate from a limited number of research groups and model systems, its apparent consistency may overstate the true effect.

## 7. Weight Loss Versus Diet Quality: Disentangling the Drivers of Immune Recovery

Most human dietary studies modify several interrelated exposures simultaneously [93]. Energy deficit reduces adipocyte size and lipid flux; weight loss changes endocrine and mechanical signals; diet composition alters fatty-acid, carbohydrate and micronutrient exposure; and behavioural interventions often increase physical activity [98]. When inflammation falls after a lifestyle programme, attributing the change specifically to food quality is therefore difficult [93]. Hypocaloric studies in adipose tissue have repeatedly shown that the magnitude of weight loss can dominate transcriptomic changes that appear superficially diet-specific [97]. A stronger design compares diets that produce similar weight loss or maintains body weight while changing dietary composition [59]. The controlled vegan-versus-ketogenic feeding study demonstrates that sharply different dietary patterns can generate distinct immune signatures over a short interval [59]. It did not enroll a post-obesity population or test persistence after washout [59]. Conversely, calorie-restriction trials can isolate energy deficit more effectively but often lack deep tissue immunophenotyping [100,115,116]. These complementary designs show why no single existing trial answers the reversal question [116].

Adipose depot is another source of heterogeneity [12]. Visceral and subcutaneous fat differ in cellular composition, vascular drainage and associations with metabolic risk, and omega-3 supplementation can produce depot-specific inflammatory responses [8,12,129,130]. A blood biomarker may therefore improve while a residual program remains in visceral tissue, or a subcutaneous biopsy may miss a biologically relevant change elsewhere [130]. Future mechanistic nutrition trials should prespecify the biological compartment that defines reversal rather than treating peripheral blood as a universal proxy [130]. Weight stability after the intervention is equally important [108]. Active weight loss involves lipolysis and transient immune-cell trafficking, whereas a weight-stable post-loss phase is more suitable for testing persistent memory [95,106,107,108]. Studies of weight regain further show that immune phenotypes can re-emerge or intensify during renewed obesogenic exposure [106]. A rigorous nutritional trial should therefore separate induction, weight-loss, stabilization and re-challenge phases rather than sampling only before and immediately after the intervention [108].

Finally, treatment context is changing [15,17]. Modern obesity management increasingly combines dietary care with surgery or pharmacotherapy, and discontinuation can produce renewed positive energy balance and weight regain [135]. The immunometabolic consequences of these trajectories may differ from those of diet-only weight cycling [135]. The central analytical task is therefore not to rank diets by anti-inflammatory biomarker change, but to identify which component of an intervention is associated with change in a persistent cellular state, in which compartment, and for how long [18,135]. A useful analytical framework therefore requires at least four separable exposures: current energy balance, current diet composition, current adiposity and prior obesity [8,97,100]. Current energy balance affects lipolysis and nutrient flux within days [95]. Diet composition changes lipid, carbohydrate and microbial substrates [95]. Adiposity determines endocrine and mechanical load [95]. Prior obesity captures the possibility of persistent cellular or tissue programs [95]. Studies that measure only baseline and final body weight collapse these exposures into one variable and cannot identify which factor is most closely associated with immune recovery [95].

The comparison group is equally important [16,17,18]. Lean participants who have never had obesity are not interchangeable with weight-reduced participants, even when body mass index is similar, because age, fat distribution, medication exposure and duration of metabolic disease can differ [18]. A stronger design would include a never-obese reference group, an obesity group, and a weight-reduced group matched as closely as possible on current adiposity, followed prospectively [18]. This three-state comparison directly tests whether prior obesity adds explanatory information beyond current body size [18]. Diet quality should then be manipulated within the weight-reduced state [51,122]. Weight-maintaining feeding can test whether replacing saturated with unsaturated fat, increasing fermentable fibre or changing overall dietary pattern alters residual immune programs without the confounding effect of further fat-mass loss [122]. Conversely, matched-weight-loss interventions can test whether two diets that produce similar adiposity changes generate different immune recovery [129]. Both approaches are more informative than comparing inflammatory biomarkers across diets that produce substantially different weight loss [40,45,129].

Temporal sequencing can strengthen mechanistic inference [35]. If a dietary change precedes a shift in immune-cell metabolism, which precedes a change in chromatin state, which then predicts a different response to re-challenge, the case for nutritional reprogramming becomes substantially stronger [29]. Cross-sectional correlations cannot establish that sequence [35]. Repeated sampling may therefore be more valuable than simply enlarging sample size when the primary question concerns memory and reversibility [26,29,31,35]. Finally, clinically meaningful outcomes should remain connected to mechanism [112]. Persistent cellular differences matter most if they predict insulin resistance, weight regain, cardiovascular events, infection response or treatment durability [98,108,112]. Mechanistic studies should therefore be nested within cohorts with sufficiently long follow-up, allowing molecular states after weight loss to be tested as predictors rather than treated as endpoints of intrinsic importance [112]. Without that link, immunometabolic memory risks becoming an elegant molecular description with uncertain clinical consequence [18].

One particularly informative strategy would combine a controlled weight-loss phase with a subsequent weight-maintenance feeding period in which diet composition is randomized [97,100,122,129]. This design would first reduce adiposity and then test whether nutritional quality modifies the residual immune state while body weight is held relatively stable [129]. Repeated adipose and blood sampling before weight loss, after stabilization and after dietary randomization would provide a direct estimate of how much recovery is attributable to adiposity reduction and how much can be shifted further by nutrition [122]. A second strategy would exploit within-person crossover feeding after weight stabilization [45,132]. Short, carefully controlled exposures to diets that differ in fat quality, fermentable fibre or ketogenic potential could quantify immediate immune-metabolic responsiveness while minimizing between-person confounding [51,59]. A washout period followed by ex vivo stimulation could then test whether any induced change persists [132]. Such a design would not prove long-term clinical benefit, but it would provide a much cleaner experimental test of nutritional plasticity than uncontrolled lifestyle cohorts [132].

These designs also help separate prevention from reversal [35]. If a healthier diet reduces inflammatory responsiveness only while it is consumed, the effect is suppression [35]. If the altered response persists after washout, it suggests reprogramming [35]. If a post-obesity response becomes indistinguishable from a never-obese reference and remains so during re-challenge, the evidence approaches functional resetting [35]. This stepwise framework converts the broad question of whether nutrition reverses memory into a series of experimentally testable contrasts [23,24,31,35].

## 8. Evidence Gaps and Criteria Required to Demonstrate Reversal of Immune Memory

The evidence base is markedly asymmetric [23,32,35,43,104,106]. In vitro systems provide precise control over stimulus, rest and re-challenge and therefore define trained-immunity mechanisms well, but they cannot reproduce adipose tissue architecture or long-term weight dynamics [23,43]. Animal models allow marrow, adipose tissue and repeated dietary transitions to be studied together and currently provide the strongest causal evidence for obesogenic memory [32,104,106]. Human observational studies establish relevance but are vulnerable to residual confounding, while human mechanistic interventions offer stronger inference yet remain few and usually lack a standardized post-weight-loss challenge [35,43]. A claim of reversal should satisfy more than biomarker improvement. At minimum, the initiating obesogenic exposure should be removed; the participant or model should enter a stable post-weight-loss state; a previously altered cell or tissue program should move toward an appropriate never-obese reference; and the change should persist beyond the immediate intervention. Stronger evidence would include restoration of cellular function, metabolic flux and chromatin state, with concordant responses to a controlled secondary challenge. Supplementary Table S5 formalizes these distinctions so that future studies do not label suppression as erasure.

The first major gap is human tissue access [17]. Repeated visceral adipose biopsies and bone-marrow sampling are difficult, so most intervention studies rely on blood or subcutaneous adipose tissue [17]. Single-cell and spatial methods can partly compensate by extracting more information from limited samples, but cross-tissue concordance remains uncertain [12,13,14]. Studies that obtain tissue during staged clinical procedures, or combine subcutaneous biopsies with peripheral immune-cell assays and circulating metabolomics, could improve mechanistic resolution without requiring invasive sampling solely for research [14]. The second gap is temporal resolution [108]. A single post-intervention time point cannot distinguish delayed recovery from durable memory [16,18,35,108]. Sampling during active weight loss, after several months of weight stability, and after longer follow-up would reveal whether persistent states decay spontaneously [108]. Re-challenge does not need to mean deliberate weight regain [35]. Standardized mixed-meal tests, short controlled feeding periods, vaccination, or ex vivo stimulation can probe whether immune responses remain primed while avoiding ethically problematic induction of obesity [35].

The third gap concerns attribution of observed effects specifically to nutrition [40]. Trials should compare energy deficit with weight-maintaining dietary substitution or use factorial designs that vary energy intake and diet quality separately [125]. Deep phenotyping should include cell composition, cytokine capacity after ex vivo stimulation, mitochondrial and glycolytic function, targeted metabolomics, chromatin accessibility or DNA methylation, and relevant adipose histology [29]. Because no single molecular mark defines immunometabolic memory, concordance across domains should matter more than isolated statistical significance [26,31]. The fourth gap is heterogeneity [8,10,100]. Sex, age, menopause, diabetes, adipose distribution, duration of obesity, magnitude and modality of weight loss, and previous weight cycling can all modify immune state [111]. Human studies commonly treat these factors as covariates rather than possible effect modifiers [111]. Stratified designs and individual-level longitudinal trajectories may be more informative than group means for identifying who retains a persistent obesogenic imprint [111].

**Figure 3:**
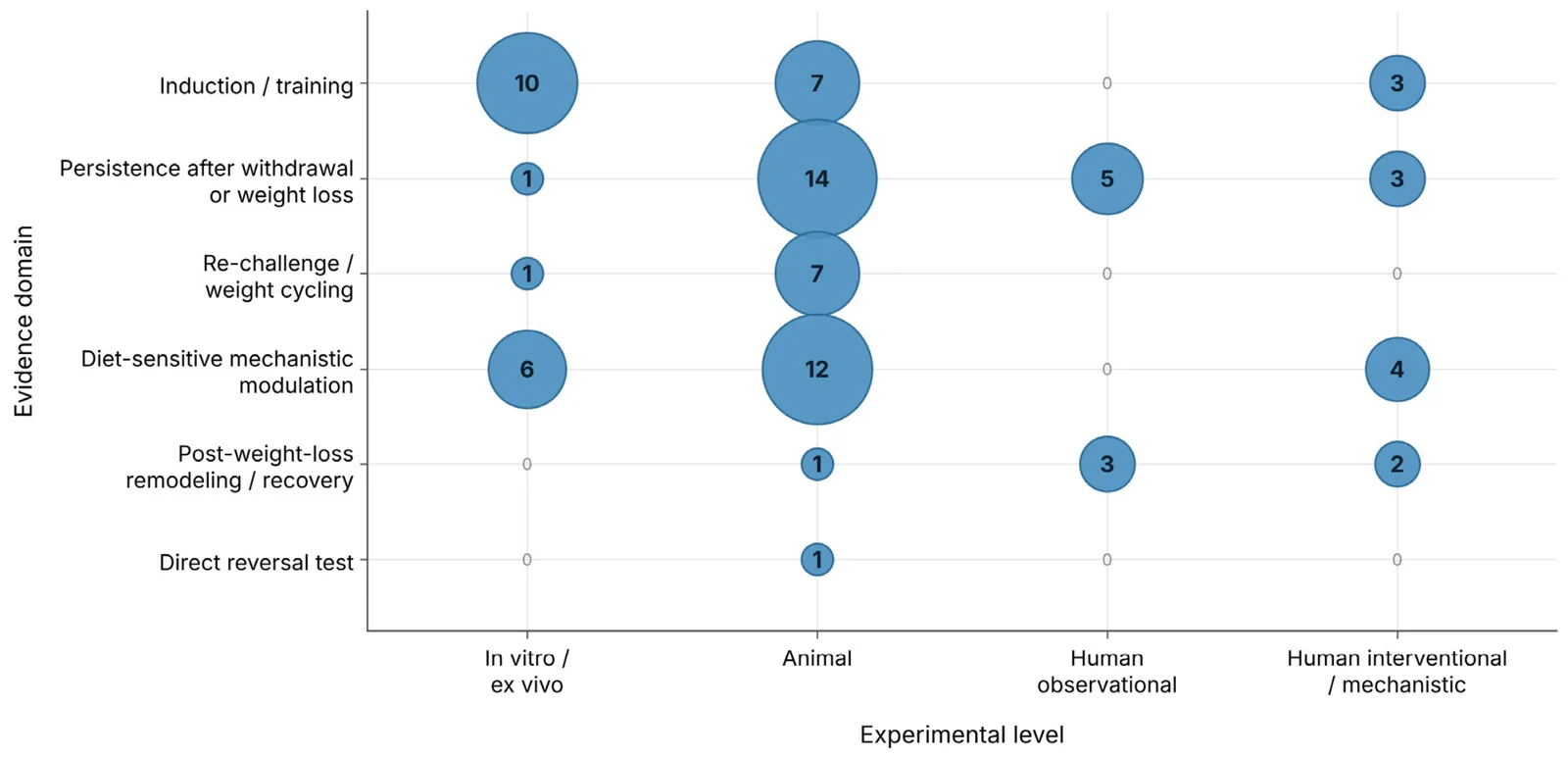
Evidence map of obesity-associated immunometabolic memory across mechanistic domains and experimental levels. Evidence is distributed across studies addressing the induction and persistence of immunometabolic alterations, responses to re-challenge or weight cycling, diet-sensitive mechanisms, post-weight-loss remodeling, and direct tests of reversal. Bubble size represents the amount of evidence within each mechanistic domain and experimental level, with the number of contributing studies shown at the center. The distribution highlights a marked imbalance between experimental and human evidence, particularly for re-challenge and direct reversal. Empty cells indicate areas in which direct evidence was not identified within the mapped evidence set and should not be interpreted as evidence of absence. Studies addressing more than one mechanistic domain may contribute to multiple cells; therefore, counts are not mutually exclusive. Created in BioRender. Reytor, C. (2026) https://BioRender.com/21qkcne.

The evidence-gap map summarizes 47 core studies directly addressing mechanistic processes relevant to immunometabolic memory. Studies were classified according to experimental level and mechanistic domain, with multiple assignments permitted when supported by distinct findings. Bubble area represents the number of studies contributing to each domain–design combination (Figure 3), while the corresponding references and classifications are provided in Supplementary Table S2.

The distribution reveals important gaps in the translation of mechanistic findings to human research, particularly regarding functional re-challenge and the reversal of established immune programming. These gaps reflect the limited availability of studies meeting the operational criteria of the evidence map rather than an absence of related investigations. Future studies could address these limitations through longitudinal assessments following weight loss, incorporating weight-stable observation periods, appropriate comparison groups, standardized metabolic challenges, and ex vivo immune-cell stimulation. Integrating functional responses with cell-type-specific transcriptional and epigenetic profiling would help distinguish persistent immune programming from transient inflammatory changes and determine whether interventions can modify established immunometabolic memory rather than merely suppress its manifestations.

Future studies should also report negative findings [17,100]. Failure to normalize a cell state after substantial weight loss can be as informative as improvement, provided the study has adequate tissue sampling and an appropriate comparator [126]. Conversely, a null epigenetic result may coexist with functional recovery, suggesting that the measured mark was not causally important [126]. A critical field should avoid treating persistence as inherently pathological or reversibility as inherently beneficial; some durable adaptations may protect tissue integrity during repeated metabolic stress [87]. The clinical endpoint remains uncertain [108]. Persistent immune or epigenetic features may contribute to weight regain, insulin resistance, cardiovascular risk or altered host defense, but causal evidence is strongest in animal models [16,18,19]. Human cohorts that link post-weight-loss molecular states to subsequent clinical trajectories are needed before immunometabolic memory becomes a therapeutic target rather than a mechanistic concept [108]. Until then, the appropriate conclusion is not that obesity permanently damages immunity, but that biological recovery after obesity appears multidimensional and may proceed on a different timescale from weight change [108].

A practical research agenda follows from these limitations. Short controlled-feeding studies are well suited to define immediate pathway responsiveness; longer weight-loss interventions can test tissue remodeling; weight-stable follow-up can determine persistence; and ethically acceptable re-challenge can test function [106]. Integrating these phases within one protocol would transform a descriptive before–after study into a direct test of memory [26,27,35]. Multi-omic measurements should be prespecified around a small number of mechanistic hypotheses rather than collected indiscriminately, because high-dimensional data cannot compensate for an intervention that lacks the temporal structure needed to establish persistence [106].

The evidence map illustrates the distribution of mechanistic research rather than a hierarchy of evidence strength. Animal studies predominate in domains requiring longitudinal tissue sampling, stimulus withdrawal, and controlled re-challenge, whereas human investigations primarily address immune remodelling and nutritional modulation. Notably, no studies meeting the operational criteria of the core evidence set populated the human re-challenge or direct-reversal categories. Although emerging human evidence demonstrates persistent obesity-associated alterations across different cellular populations and tissue compartments [113], the available findings remain limited in scope and mechanistic resolution. These gaps highlight the need for longitudinal human studies capable of distinguishing persistent cellular programming from transient inflammatory responses and establishing whether obesity-associated immunometabolic alterations can be functionally reversed.

An independent analysis by Corrao et al. used artificial intelligence-based semantic mapping to characterize the conceptual organization of obesity-related literature, identifying three interconnected domains: inflammatory adipose biology, adipose remodelling and chronic dysfunction, and stress-triggered immune persistence [148]. The authors interpreted this organization as compatible with obesogenic memory conceptualized as biological hysteresis, while acknowledging that semantic convergence does not establish mechanistic causality. Although conceptually complementary, the two approaches address different dimensions of the literature. Their analysis identifies thematic relationships across publications, whereas our evidence map classifies primary studies according to experimental level and mechanistic domain. This distinction is important because conceptual coherence does not establish the strength of the underlying experimental evidence. Together, these approaches provide complementary perspectives on the emerging field while emphasizing the need for direct human investigations of immunometabolic persistence, re-challenge, and reversibility.

## 9. Conclusions

Obesity appears to leave a biological history that can remain detectable after weight loss, but the nature of that history differs across tissues and cell populations. Persistent adipose, immune and epigenetic states should not be interpreted as a single permanent lesion. They are better viewed as partially overlapping phenomena with different timescales, mechanisms and degrees of reversibility. The available evidence is strongest in experimental systems, where stimulus withdrawal and re-challenge can be controlled. Human studies increasingly support incomplete molecular normalization after weight loss, but they do not yet establish the duration, functional consequences or clinical importance of every residual signal.

Nutrition can modify many pathways implicated in obesity-associated immune dysfunction, including substrate sensing, inflammatory signaling, mitochondrial metabolism and microbiota-derived signaling. That capacity does not prove that nutrition can erase an established immunometabolic program. A lower inflammatory biomarker level, reduced immune-cell abundance or improved insulin sensitivity may reflect suppression or remodeling rather than durable resetting. Claims of reversal therefore require evidence that the altered state has normalized after the original exposure has ended and remains normalized during follow-up or renewed challenge.

The most informative future studies will separate energy deficit from diet composition, include a weight-stable post-loss phase and integrate cellular function with transcriptional, epigenetic and metabolic measurements. Studies that compare weight-reduced individuals with never-obese participants and that incorporate controlled re-challenge would provide particularly strong tests of immunometabolic hysteresis. Until such evidence accumulates, obesity-associated immunometabolic memory is best treated as a useful mechanistic framework rather than an established clinical entity. Its value lies in sharpening the distinction between reduced inflammation, persistent biological remodeling and genuine reversal, and in defining the experiments needed to distinguish among them.

## Figures and Tables

**Table 1:** Studies that directly support or challenge the concept of obesity-associated immunometabolic memory.

Study	Species/Evidence Level	Model/Design	Memory-Related Observation	Critical Interpretation
Christ et al., 2018 [32]	Mouse; preclinical in vivo	Mouse Western diet; progenitor profiling	Persistent NLRP3-dependent reprogramming of myeloid progenitors after diet withdrawal	Strong diet-to-innate-memory mechanism; animal model
Zamarron et al., 2017 [101]	Mouse; preclinical in vivo	Formerly obese mice	Macrophage proliferation sustained adipose inflammation after weight loss	Persistence without complete metabolic equivalence
Zou et al., 2018 [105]	Mouse; preclinical in vivo with adoptive transfer	Mouse gain–loss–regain; adoptive transfer	CD4+ T cells transferred an obesity-history phenotype	Functional adaptive-memory evidence; uncertain human translation
Blaszczak et al., 2020 [104]	Mouse; preclinical in vivo	Formerly obese mice	Immune and adipocyte abnormalities persisted after body-weight normalization	Supports distributed tissue memory
Cottam et al., 2022 [106]	Mouse; preclinical in vivo	Mouse CITE-seq across obesity, weight loss, regain	Obesity-associated immune phenotypes persisted and intensified with regain	Strong systems-level persistence
Caslin et al., 2023 [107]	Mouse and murine cells; preclinical	Mouse weight cycling	Adipose macrophages displayed innate-memory features	Supports trained-immunity interpretation
Hinte et al., 2024 [16]	Human and mouse; human longitudinal plus preclinical causal arm	Human adipose longitudinal + mouse epigenetics	Persistent human adipose transcriptional signatures; adipocyte epigenetic memory in mice	Key human relevance; causality strongest in mouse arm
Miranda et al., 2025 [17]	Human; observational longitudinal	Human spatial/single-nucleus atlas before/after weight loss	Selective remodeling with incomplete normalization of some adipose states	Demonstrates nonuniform recovery, not necessarily memory in every lineage
Niven et al., 2026 [18]	Human; observational longitudinal	Human CD4+ T-cell methylome after weight loss	Obesity-associated DNA methylation persisted after weight loss	Human adaptive persistence; clinical consequences require confirmation
Garcia et al., 2026 [108]	Mouse; preclinical interventional (gene deletion)	Mouse weight cycling; CD70-CD27 disruption	Blocking memory formation mitigated exaggerated metabolic response	One of the strongest mechanistic tests of memory relevance
van Tuijl et al., 2025 [113]	Human; observational case–cohort plus ex vivo experiments	Adults before and six months after metabolic bariatric surgery, with normal-weight comparison group	Monocyte hyperresponsiveness only partially reverted despite normalization of leukocyte counts and hs-CRP	Direct human evidence of residual innate training; adipose tissue implicated as the inducing signal

Abbreviations: CD4, cluster of differentiation 4; CD27, cluster of differentiation 27; CD70, cluster of differentiation 70; CITE-seq, cellular indexing of transcriptomes and epitopes by sequencing;DNA, deoxyribonucleic acid; hs-CRP, high-sensitivity C-reactive protein; NLRP3, NOD-, LRR- and pyrin domain-containing protein 3. Note: Table 1 presents selected studies from the core evidence set (Section 2; Supplementary Table S3), highlighting evidence of persistent immunometabolic alterations following stimulus withdrawal, functional consequences of prior exposure, and incomplete normalization after weight loss. Studies reporting inflammation exclusively during established obesity were not considered. Species and experimental design are specified to contextualize the findings and their translational relevance.

## Data Availability

No new data were created or analyzed in this study. The evidence supporting this review is derived from the published literature cited in the manuscript. The search strategies and study-level information used to construct the evidence map are provided in the Supplementary Materials.

## Supplementary Materials

The following supporting information can be downloaded at: https://www.mdpi.com/article/10.3390/ijms27219666/s1.
