# Supplementary material for: Immunometabolic Memory in Obesity: Nutritional Drivers, Persistence, and Reversibility
Source: Int J Mol Sci. 2026 Oct 28;27(21):9666. doi: 10.3390/ijms27219666 (PMC13762619; doi:10.3390/ijms27219666)
Supplement: Supplementary file 1 [file ijms-27-09666-s001.zip › ijms-4632366-supplementary.pdf]

Supplementary Material

Immunometabolic Memory in Obesity: Nutritional Drivers, Persistence, and Reversibility

Table S1. Structured search strategy

Source	Date last searched	Search approach / strategy
PubMed/MEDLINE	6 September 2026	(("Obesity"[Mesh] OR "Adipose Tissue"[Mesh] OR "Weight Loss"[Mesh] OR obesity[tiab] OR adiposity[tiab] OR "adipose tissue"[tiab] OR "weight loss"[tiab] OR "weight cycling"[tiab]) AND ("Immunity, Innate"[Mesh] OR "Immunologic Memory"[Mesh] OR "Epigenesis, Genetic"[Mesh] OR "Macrophages"[Mesh] OR "Monocytes"[Mesh] OR "Lymphocytes"[Mesh] OR "Hematopoietic Stem Cells"[Mesh] OR "trained immunity"[tiab] OR "innate immune memory"[tiab] OR "immune memory"[tiab] OR immunometabol*[tiab] OR "metabolic memory"[tiab] OR "epigenetic memory"[tiab] OR "epigenetic reprogramming"[tiab]) AND ("Diet"[Mesh] OR "Diet Therapy"[Mesh] OR "Caloric Restriction"[Mesh] OR diet*[tiab] OR nutrition*[tiab] OR nutrient*[tiab] OR "dietary pattern*"[tiab] OR "calorie restriction"[tiab] OR "caloric restriction"[tiab] OR "energy restriction"[tiab]))

Cochrane Library	6 September 2026	#1 MeSH descriptor: [Obesity] explode all trees<br>#2 MeSH descriptor: [Adipose Tissue] explode all trees<br>#3 MeSH descriptor: [Weight Loss] explode all trees<br>#4 (obesity or adiposity or "adipose tissue" or "weight loss" or "weight cycling"):ti,ab,kw<br>#5 #1 or #2 or #3 or #4<br>#6 MeSH descriptor: [Immunity, Innate] explode all trees<br>#7 MeSH descriptor: [Immunologic Memory] explode all trees<br>#8 MeSH descriptor: [Epigenesis, Genetic] explode all trees<br>#9 MeSH descriptor: [Macrophages] explode all trees<br>#10 MeSH descriptor: [Monocytes] explode all trees<br>#11 ("trained immunity" or "innate immune memory" or "immune memory" or immunometabol* or "metabolic memory" or "epigenetic memory" or "epigenetic reprogramming"):ti,ab,kw<br>#12 #6 or #7 or #8 or #9 or #10 or #11<br>#13 MeSH descriptor: [Diet] explode all trees<br>#14 MeSH descriptor: [Caloric Restriction] explode all trees<br>#15 (diet* or nutrition* or nutrient* or "dietary pattern*" or "calorie restriction" or "caloric restriction" or "energy restriction"):ti,ab,kw<br>#16 #13 or #14 or #15<br>#17 #5 and #12 and #16

Epistemonikos	6 September 2026	(title:(obesity OR adiposity OR "adipose tissue" OR "weight loss" OR "weight cycling") OR abstract:(obesity OR adiposity OR "adipose tissue" OR "weight loss" OR "weight cycling")) AND (title:("trained immunity" OR "innate immune memory" OR "immune memory" OR immunometabol* OR "metabolic memory" OR "epigenetic memory" OR "epigenetic reprogramming") OR abstract:("trained immunity" OR "innate immune memory" OR "immune memory" OR immunometabol* OR "metabolic memory" OR "epigenetic memory" OR "epigenetic reprogramming")) AND (title:(diet* OR nutrition* OR nutrient* OR "dietary pattern" OR "calorie restriction" OR "caloric restriction" OR "energy restriction") OR abstract:(diet* OR nutrition* OR nutrient* OR "dietary pattern" OR "calorie restriction" OR "caloric restriction" OR "energy restriction"))
Citation tracking	6 September 2026	Backward reference-list screening of eligible studies and relevant reviews, complemented by forward citation tracking of pivotal mechanistic and longitudinal studies.

No date or language restrictions were applied to study eligibility. Database syntax was adapted to the functionality of each source. PubMed/MEDLINE and the Cochrane Library incorporated controlled vocabulary and free-text terms; Epistemonikos used Boolean free-text searching. Studies identified during peer review were incorporated into the synthesis and, where they met the criteria for the core evidence set, into the evidence map.

Figure S1. Synthesis framework

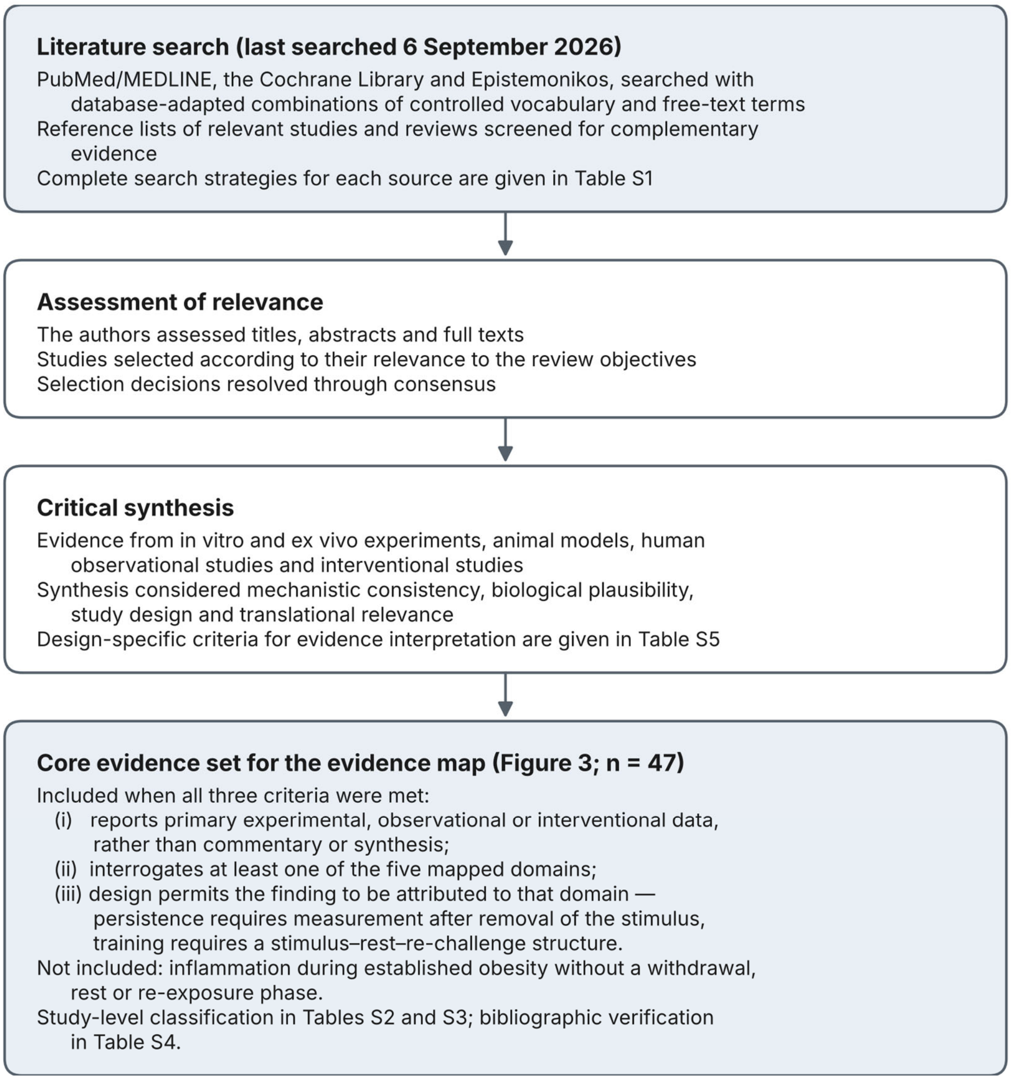

Figure S1. Synthesis framework. The diagram summarizes the literature search, the assessment of relevance, the critical synthesis, and the criteria by which the core evidence set underlying the evidence map in Figure 3 was defined.

Table S2. Coding framework for the visual evidence map

The visual evidence map in Figure 3 summarizes the 47-study mechanistic core across predefined evidence domains and experimental levels. Each study can contribute to more than one domain or level when distinct findings support multiple classifications; therefore, cell counts are not additive. The references below correspond to the revised manuscript.

Evidence domain	Experimental level	n	Contributing manuscript references
Induction / training	In vitro / ex vivo	10	[25, 26, 27, 28, 29, 30, 31, 42, 43, 113]

Induction / training	Animal	7	[25, 27, 32, 33, 34, 37, 42]
Induction / training	Human observational	0	None
Induction / training	Human interventional / mechanistic	3	[28, 35, 36]
Persistence after withdrawal / weight loss	In vitro / ex vivo	1	[42]
Persistence after withdrawal / weight loss	Animal	14	[16, 32, 33, 34, 42, 57, 95, 101, 102, 103, 104, 105, 106, 107]
Persistence after withdrawal / weight loss	Human observational	5	[16, 17, 18, 99, 113]
Persistence after withdrawal / weight loss	Human interventional / mechanistic	3	[35, 36, 100]
Re-challenge / weight cycling	In vitro / ex vivo	1	[25]
Re-challenge / weight cycling	Animal	7	[25, 57, 104, 105, 106, 107, 108]
Re-challenge / weight cycling	Human observational	0	None
Re-challenge / weight cycling	Human interventional / mechanistic	0	None
Diet-sensitive mechanistic modulation	In vitro / ex vivo	6	[39, 40, 44, 45, 46, 52]
Diet-sensitive mechanistic modulation	Animal	12	[38, 39, 40, 44, 45, 46, 47, 51, 52, 54, 58, 103]
Diet-sensitive mechanistic modulation	Human observational	0	None
Diet-sensitive mechanistic modulation	Human interventional / mechanistic	4	[58, 59, 122, 123]
Post-weight-loss remodeling / recovery	In vitro / ex vivo	0	None
Post-weight-loss remodeling / recovery	Animal	1	[95]
Post-weight-loss remodeling / recovery	Human observational	3	[17, 92, 99]
Post-weight-loss remodeling / recovery	Human interventional / mechanistic	2	[91, 100]

Direct reversal test	In vitro / ex vivo	0	None
Direct reversal test	Animal	1	[108]
Direct reversal test	Human observational	0	None
Direct reversal test	Human interventional / mechanistic	0	None

Table S3. Core evidence set used for the visual evidence map (n = 47)

Ref.	Study	Evidence level	Primary mechanism / target	Map domain
16	Hinte et al., 2024	Human longitudinal / animal	Adipose epigenetic memory	Persistence
17	Miranda et al., 2025	Human longitudinal	Selective adipose-niche remodeling	Persistence/recovery
18	Niven et al., 2026	Human longitudinal	CD4 T-cell methylation memory	Persistence
25	Quintin et al., 2012	In vitro / animal	Canonical trained immunity	Induction; rechallenge
26	Cheng et al., 2014	In vitro / animal	Glycolytic training	Induction
27	Saeed et al., 2014	In vitro	Epigenetic programming	Induction
28	Arts et al., 2016	Human / in vitro	BCG immunometabolism	Induction
29	Arts et al., 2016	In vitro	Fumarate–epigenetic coupling	Induction
30	Bekkering et al., 2014	Human in vitro	oxLDL training	Induction
31	Bekkering et al., 2018	Human in vitro	Mevalonate training	Induction
32	Christ et al., 2018	Animal	Western-diet training	Induction; persistence
33	Mitroulis et al., 2018	Animal	Myeloid progenitor memory	Induction; persistence
34	Kaufmann et al., 2018	Animal	HSC training	Induction; persistence
35	Cirovic et al., 2020	Human interventional	HSPC training after BCG	Induction; persistence
36	Sun et al., 2024	Human interventional	Bone-marrow epigenetic remodeling	Induction; persistence

37	Singer et al., 2014	Animal	Obesity-induced myelopoiesis	Induction
38	Vandanmagsar et al., 2011	Animal	NLRP3 obesity inflammation	Diet-sensitive mechanism
39	Wen et al., 2011	In vitro / animal	Fatty-acid NLRP3 signaling	Diet-sensitive mechanism
40	Lancaster et al., 2018	In vitro / animal	Lipid-TLR4 metabolic reprogramming	Diet-sensitive mechanism
42	Thiem et al., 2021	Human in vitro / animal	Hyperglycemic innate memory	Induction; persistence
43	Topham et al., 2026	Human in vitro	Obesity factors and beta-glucan priming	Induction
44	Pan et al., 2020	In vitro / animal	Oat beta-glucan training	Diet-sensitive mechanism
45	Oh et al., 2010	In vitro / animal	Omega-3 / FFAR4	Diet-sensitive mechanism
46	Youm et al., 2015	In vitro / animal	BHB–NLRP3 inhibition	Diet-sensitive mechanism
47	Hellmann et al., 2011	Animal	Resolvin D1	Diet-sensitive mechanism
51	Smith et al., 2013	Animal	SCFA–Treg axis	Diet-sensitive mechanism
52	Furusawa et al., 2013	In vitro / animal	Butyrate epigenetic Treg induction	Diet-sensitive mechanism
54	Cani et al., 2007	Animal	Metabolic endotoxemia	Diet-sensitive mechanism
57	Thaiss et al., 2016	Animal	Persistent post-diet microbiome	Persistence; rechallenge
58	Ang et al., 2020	Human interventional / animal	Ketogenic diet microbiome–Th17	Dietary modulation
59	Link et al., 2024	Human interventional	Vegan vs ketogenic immune signatures	Dietary modulation
91	Clément et al., 2004	Human interventional	VLCD adipose transcriptome	Reversibility/remodeling
92	Cancello et al., 2005	Human longitudinal	Surgery-induced macrophage reduction	Reversibility/remodeling
95	Kosteli et al., 2010	Animal	Weight-loss immune dynamics	Persistence/recovery

99	Hagman et al., 2017	Human longitudinal	Post-bariatric adipose immunity	Persistence/recovery
100	Sbierski-Kind et al., 2020	Human randomized	Calorie restriction adipose immunity	Persistence/recovery
101	Zamarron et al., 2017	Animal	Former-obesity macrophage persistence	Persistence
102	Surendar et al., 2020	Animal	CD8 inflammation during weight loss	Persistence
103	Rodrigues et al., 2020	Animal	High-fat calorie restriction	Persistence; dietary modulation
104	Blaszczak et al., 2020	Animal	Obesogenic memory	Persistence; rechallenge
105	Zou et al., 2018	Animal	CD4 T-cell obesity memory	Persistence; rechallenge
106	Cottam et al., 2022	Animal multiomics	Weight-loss/regain immune persistence	Persistence; rechallenge
107	Caslin et al., 2023	Animal	ATM trained memory in weight cycling	Persistence; rechallenge
108	Garcia et al., 2026	Animal	T-cell memory disruption	Rechallenge; reversal test
113	van Tuijl et al., 2025	Human observational / ex vivo	Trained monocyte phenotype after bariatric surgery	Persistence; induction (ex vivo)
122	Bakker et al., 2023	Human randomized	Omega-3 adipose inflammation	Dietary modulation
123	Fisk et al., 2022	Human randomized	Omega-3 adipose inflammation	Dietary modulation

ATM, adipose tissue macrophage; BCG, Bacille Calmette–Guérin; BHB, beta-hydroxybutyrate; FFAR4, free fatty acid receptor 4; HSC, hematopoietic stem cell; HSPC, hematopoietic stem and progenitor cell; NLRP3, NOD-, LRR- and pyrin domain-containing protein 3; oxLDL, oxidized low-density lipoprotein; SCFA, short-chain fatty acid; Treg, regulatory T cell; VLCD, very-low-calorie diet. Reference numbers correspond to the revised manuscript and are ordered ascendingly. The numbering is non-contiguous because the core evidence set is a subset of the full reference list, which also contains background, conceptual and methodological sources. Entries 26 and 27 have been corrected: reference 26 is Cheng et al., 2014 and reference 27 is Saeed et al., 2014.

Table S4. Bibliographic validation log for the core evidence set

Reference	Validation source	Identifier available in manuscript	Role in synthesis
[16] Hinte et al., 2024	PubMed/MEDLINE or publisher record	doi:10.1038/s41586-024-08165-7	Persistence

[17] Miranda et al., 2025	PubMed/MEDLINE or publisher record	doi:10.1038/s41586-025-09233-2	Persistence/recovery
[18] Niven et al., 2026	PubMed/MEDLINE or publisher record	doi:10.1038/s44319-026-00765-w	Persistence
[25] Quintin et al., 2012	PubMed/MEDLINE or publisher record	doi:10.1016/j.chom.2012.06.006	Induction; rechallenge
[26] Cheng et al., 2014	PubMed/MEDLINE or publisher record	doi:10.1126/science.1250684	Induction
[27] Saeed et al., 2014	PubMed/MEDLINE or publisher record	doi:10.1126/science.1251086	Induction
[28] Arts et al., 2016	PubMed/MEDLINE or publisher record	doi:10.1016/j.celrep.2016.11.011	Induction
[29] Arts et al., 2016	PubMed/MEDLINE or publisher record	doi:10.1016/j.cmet.2016.10.008	Induction
[30] Bekkering et al., 2014	PubMed/MEDLINE or publisher record	doi:10.1161/ATVBAHA.114.303887	Induction
[31] Bekkering et al., 2018	PubMed/MEDLINE or publisher record	doi:10.1016/j.cell.2017.11.025	Induction
[32] Christ et al., 2018	PubMed/MEDLINE or publisher record	doi:10.1016/j.cell.2017.12.013	Induction; persistence
[33] Mitroulis et al., 2018	PubMed/MEDLINE or publisher record	doi:10.1016/j.cell.2017.11.034	Induction; persistence
[34] Kaufmann et al., 2018	PubMed/MEDLINE or publisher record	doi:10.1016/j.cell.2017.12.031	Induction; persistence
[35] Cirovic et al., 2020	PubMed/MEDLINE or publisher record	doi:10.1016/j.chom.2020.05.014	Induction; persistence
[36] Sun et al., 2024	PubMed/MEDLINE or publisher record	doi:10.1016/j.immuni.2024.07.021	Induction; persistence
[37] Singer et al., 2014	PubMed/MEDLINE or publisher record	doi:10.1016/j.molmet.2014.06.005	Induction
[38] Vandanmagsar et al., 2011	PubMed/MEDLINE or publisher record	doi:10.1038/nm.2279	Diet-sensitive mechanism
[39] Wen et al., 2011	PubMed/MEDLINE or publisher record	doi:10.1038/ni.2022	Diet-sensitive mechanism
[40] Lancaster et al., 2018	PubMed/MEDLINE or publisher record	doi:10.1016/j.cmet.2018.03.014	Diet-sensitive mechanism
[42] Thiem et al., 2021	PubMed/MEDLINE or publisher record	doi:10.4049/jimmunol.1901348	Induction; persistence
[43] Topham et al., 2026	PubMed/MEDLINE or publisher record	doi:10.3389/fimmu.2026.1825431	Induction

[44] Pan et al., 2020	PubMed/MEDLINE or publisher record	doi:10.1007/s10753-020-01211-2	Diet-sensitive mechanism
[45] Oh et al., 2010	PubMed/MEDLINE or publisher record	doi:10.1016/j.cell.2010.07.041	Diet-sensitive mechanism
[46] Youm et al., 2015	PubMed/MEDLINE or publisher record	doi:10.1038/nm.3804	Diet-sensitive mechanism
[47] Hellmann et al., 2011	PubMed/MEDLINE or publisher record	doi:10.1096/fj.10-178657	Diet-sensitive mechanism
[51] Smith et al., 2013	PubMed/MEDLINE or publisher record	doi:10.1126/science.1241165	Diet-sensitive mechanism
[52] Furusawa et al., 2013	PubMed/MEDLINE or publisher record	doi:10.1038/nature12721	Diet-sensitive mechanism
[54] Cani et al., 2007	PubMed/MEDLINE or publisher record	doi:10.2337/db06-1491	Diet-sensitive mechanism
[57] Thaiss et al., 2016	PubMed/MEDLINE or publisher record	doi:10.1038/nature20796	Persistence; rechallenge
[58] Ang et al., 2020	PubMed/MEDLINE or publisher record	doi:10.1016/j.cell.2020.04.027	Dietary modulation
[59] Link et al., 2024	PubMed/MEDLINE or publisher record	doi:10.1038/s41591-023-02761-2	Dietary modulation
[91] Clément et al., 2004	PubMed/MEDLINE or publisher record	doi:10.1096/fj.04-2204com	Reversibility/remodeling
[92] Cancello et al., 2005	PubMed/MEDLINE or publisher record	doi:10.2337/diabetes.54.8.2277	Reversibility/remodeling
[95] Kosteli et al., 2010	PubMed/MEDLINE or publisher record	doi:10.1172/JCI42845	Persistence/recovery
[99] Hagman et al., 2017	PubMed/MEDLINE or publisher record	doi:10.1016/j.metabol.2017.01.030	Persistence/recovery
[100] Sbierski-Kind et al., 2020	PubMed/MEDLINE or publisher record	doi:10.4049/jimmunol.2000108	Persistence/recovery
[101] Zamarron et al., 2017	PubMed/MEDLINE or publisher record	doi:10.2337/db16-0500	Persistence
[102] Surendar et al., 2020	PubMed/MEDLINE or publisher record	doi:10.3389/fendo.2020.00257	Persistence
[103] Rodrigues et al., 2020	PubMed/MEDLINE or publisher record	doi:10.1139/apnm-2020-0220	Persistence; dietary modulation
[104] Blaszczak et al., 2020	PubMed/MEDLINE or publisher record	doi:10.20900/immunometab20200023	Persistence; rechallenge
[105] Zou et al., 2018	PubMed/MEDLINE or publisher record	doi:10.1038/cmi.2017.36	Persistence; rechallenge

[106] Cottam et al., 2022	PubMed/MEDLINE or publisher record	doi:10.1038/s41467-022-30646-4	Persistence; rechallenge
[107] Caslin et al., 2023	PubMed/MEDLINE or publisher record	doi:10.3389/fimmu.2022.984859	Persistence; rechallenge
[108] Garcia et al., 2026	PubMed/MEDLINE or publisher record	doi:10.2337/db25-0605	Rechallenge; reversal test
[113] van Tuijl et al., 2025	PubMed/MEDLINE or publisher record	doi:10.1038/s41366-025-01886-3	Persistence; induction (ex vivo)
[122] Bakker et al., 2023	PubMed/MEDLINE or publisher record	doi:10.1016/j.nut.2023.112055	Dietary modulation
[123] Fisk et al., 2022	PubMed/MEDLINE or publisher record	doi:10.1016/j.ebiom.2022.103909	Dietary modulation

Supplementary note: interpretation of evidence levels

In vitro evidence was considered mechanistically informative when a stimulus–rest–rechallenge structure or durable metabolic/epigenetic phenotype was demonstrated. Animal evidence was considered most informative when the model included obesity induction followed by diet withdrawal, weight normalization or weight cycling. Human observational evidence required a post-weight-loss or post-obesity comparison with molecular or cellular phenotyping. Human interventional/mechanistic evidence required a controlled nutritional or weight-loss intervention with immune, tissue, metabolic or epigenetic outcomes. These categories indicate design type, not formal certainty-of-evidence grades. Reference numbers correspond to the revised manuscript and are ordered ascendingly. The identifier for Caslin et al., 2023 has been corrected to the official record (Front Immunol 2023;13:984859), and the identifier for Fisk et al., 2022 is now given as a DOI.

Table S5. Operational definitions used in the critical synthesis

Concept	Operational criterion	What is insufficient	Representative evidence
Reduced inflammatory tone	Lower circulating or tissue inflammatory output during or after intervention	A decrease in CRP or cytokines alone	Diet/weight-loss studies [91–100, 115, 116, 122–130]
Cellular remodeling	Change in cell abundance, phenotype, metabolism or transcription toward a healthier reference	Weight loss without cellular measurements	Single-cell and tissue studies [12–17, 86–90]
Persistent immunometabolic alteration	Cellular/molecular difference remains after the initiating obesogenic exposure is removed	Abnormality measured during ongoing obesity	Former-obesity studies [16–18, 101–108, 111, 112]
Trained immunity	Innate cell/progenitor reprogramming persists through a rest phase and changes response to a secondary stimulus	Chronic innate activation without rest/rechallenge	Canonical and diet-induced training [23–43]
Reversal / resetting	Post-obesity state converges toward never-obese reference across durable functional and molecular endpoints	Short-term biomarker normalization	Not convincingly demonstrated for nutrition in humans

CRP, C-reactive protein. Definitions are analytical rather than diagnostic and are intended to distinguish transient anti-inflammatory effects from durable reversal of a cellular program.
